# Extended SSD VMAT treatment for total body irradiation

**DOI:** 10.1002/acm2.12519

**Published:** 2018-12-27

**Authors:** Greg Pierce, Alex Balogh, Rebecca Frederick, Deborah Gordon, Adam Yarschenko, Alana Hudson

**Affiliations:** ^1^ Department of Medical Physics Tom Baker Cancer Centre Calgary AB Canada; ^2^ Department of Physics & Astronomy University of Calgary Calgary AB Canada; ^3^ Department of Oncology University of Calgary Calgary AB Canada; ^4^ Division of Radiation Oncology Tom Baker Cancer Centre Calgary AB Canada; ^5^ Department of Radiation Therapy Tom Baker Cancer Centre Calgary AB Canada

**Keywords:** inverse planning, patient comfort, total body irradiation, volumetric modulated arc therapy

## Abstract

In this work, we develop a total body irradiation technique that utilizes arc delivery, a buildup spoiler, and inverse optimized multileaf collimator (MLC) motion to shield organs at risk. The current treatment beam model is verified to confirm its applicability at extended source‐to‐surface distance (SSD). The delivery involves 7–8 volumetric modulated arc therapy arcs delivered to the patient in the supine and prone positions. The patient is positioned at a 90° couch angle on a custom bed with a 1 cm acrylic spoiler to increase surface dose. Single‐step optimization using a patient CT scan provides enhanced dose homogeneity and limits organ at risk dose. Dosimetric data of 109 TBI patients treated with this technique is presented along with the clinical workflow. Treatment planning system (TPS) verification measurements were performed at an extended SSD of 175 cm. Measurements included: a 4‐point absolute depth‐dose curve, profiles at 1.5, 5, and 10 cm depth, absolute point‐dose measurements of an treatment field, 2D Gafchromic^®^ films at four locations, and measurements of surface dose at multiple locations of a Alderson phantom. The results of the patient DVH parameters were: Body‐5 mm D98 95.3 ± 1.5%, Body‐5 mm D2 114.0 ± 3.6%, MLD 102.8 ± 2.1%. Differences between measured and calculated absolute depth‐dose values were all <2%. Profiles at extended SSD had a maximum point difference of 1.3%. Gamma pass rates of 2D films were greater than 90% at 5%/1 mm. Surface dose measurements with film confirmed surface dose values of >90% of the prescription dose. In conclusion, the inverse optimized delivery method presented in the paper has been used to deliver homogenous dose to over 100 patients. The method provides superior patient comfort utilizing a commercial TPS. In addition, the ability to easily shield organs at risk is available through the use of MLCs.

## 1 INTRODUCTION

Total body irradiation (TBI) is an essential part of bone marrow transplant conditioning as it has been shown to eliminate residual chemotherapy‐resistant cancer cells and it provides additional immunosuppression to enhance engraftment.^1^ TBI is combined with chemotherapy to enhance dose intensity of the preparative regimen while avoiding overlapping toxicity that may occur with high‐dose multi‐drug regimens without radiation. While the goal of TBI is to deliver a homogeneous dose of radiation to the body^1^, ^2^ there is currently no consensus on the optimal technique or fractionation to deliver the prescription dose most safely and effectively.^3^, ^4^, ^5^, ^6^ Conditioning regimens that use low dose TBI generally do not result in significant side effects; however lung shielding is used to maintain an even dose throughout the body.

A variety of TBI delivery techniques have been developed. Most of these techniques involve opposing beams that are either lateral or anterior‐posterior (AP/PA) and are performed with the patient at an extended distance to limit the need for junctioning fields.^7^, ^8^ An example of a lateral technique is lateral parallel opposed pair (POP) beams with the patient under full bolus, which provides good dose homogeneity and utilizes simple dose calculation algorithms.^9^, ^10^ Drawbacks to this approach include reduced patient comfort and the limited ability to shield organs at risk without compromising dose to bones. Examples of AP/PA techniques include translating bed and multiple field techniques, such as the “mick” technique that uses multiple fields and boosts to achieve the required dose distribution.^11^, ^12^, ^13^, ^14^, ^15^, ^16^ AP/PA techniques provide the ability to shield organs at risk (usually through poured blocks) but other challenges exist. For instance, translating bed techniques require external beds custom designed to be under computer control with custom dosimetry calculations. In addition, multiple field techniques require accurate matching of field junctions in order to limit hot and cold regions during delivery to the patient.

Recently, there have been attempts to incorporate aspects of volumetrically modulated arc treatment (VMAT) into TBI treatments. Kirby et al.^17^ developed an inversely modulated pseudo‐arc technique. Multiple static beams are delivered in an arc configuration using inverse planning to develop the monitor units (MU) for each. The patient is treated in an AP/PA orientation with cerrobend blocks used to shield the lungs, and a hanging spoiler was used to increase the surface dose. Springer et al.^18^ developed a full inversely planned arc technique with the patient laying on the treatment bed at isocenter. Multiple beam isocenters are used with the patient being translated longitudinally and the inverse optimization providing smoothing at the junctions. While the dose distributions for these plans were acceptable, the planning and contouring time was significant. Polednik et al.^19^ demonstrated an ultra‐efficient modulated arc delivery method consisting of multiple consecutive modulated 5° subarcs in order to produce an homogenous dose. Jahnke et al.^20^ recently proposed single modulated sweeping arc version of this technique where the patient is treated AP/PA at extended source‐to‐surface distance (SSD) and a sweeping arc covers their entire body. The arc speed is varied to account for inverse square law (ISL) effects and to provide a homogenous dose. Organs at risk were shielded with poured blocks, and surface dose was increased with a beam spoiler. Jahnke et al. reported dose homogeneity of ±10% for a block phantom, however homogeneity was not considered for actual patients. In this work we build on this technique by modifying Jahnke et al.'s standard arcs to accommodate for SSD variation by using patient CT data. We also employ an inverse planned, single setup optimization from a commercial treatment planning system (TPS) to provide a VMAT solution (Eclipse^®^; Varian Medical Systems, Palo Alto, CA, USA) with the AAA algorithm that has been shown to perform well at extended SSD.^21^, ^22^ The optimization modifies the multi‐leaf collimator (MLC) positions to shield organs at risk and to provide dose homogeneity throughout the body, producing a personalized, deliverable treatment plan that interfaces with the record and verify system. We report on our patient experience and the measurements made to verify the accuracy of our TPS's beam model at extended SSD.

## 2 METHODS

### 2.A Patient characteristics and planning objectives

Between January 2016 and August 2017, 109 patients with an average age of 44 (range 4–70) and disease types such as, but not limited to, MDS, AML, and ALL have been treated according to the technique presented below. Four different prescriptions were used: 86 patients with 400 cGy in two fractions, 10 patients with 200 cGy in one fraction, eight patients with 300 cGy in one fraction, and six patients with 500 cGy in six fractions. Dosimetric coverage was optimized to ensure that the D98% and D2% for the entire body structure (retracted 5 mm from the skin) and the mean lung dose (MLD) were approximately equal to the prescription dose. All values are quoted in percentage of the prescription dose. Additionally, we present in vivo measurements of one fraction for five patients at eight locations, at the temple, umbilicus, lumbar back, left foot and four locations on the legs (anterior and posterior calf and thigh). Measurements were performed using microdot OSLDs and read with MicroStar^®^ reader system (Landauer, Glenwood, IL, USA).

### 2.B Treatment technique

#### 2.B.1 CT scan

**Figure 1:**
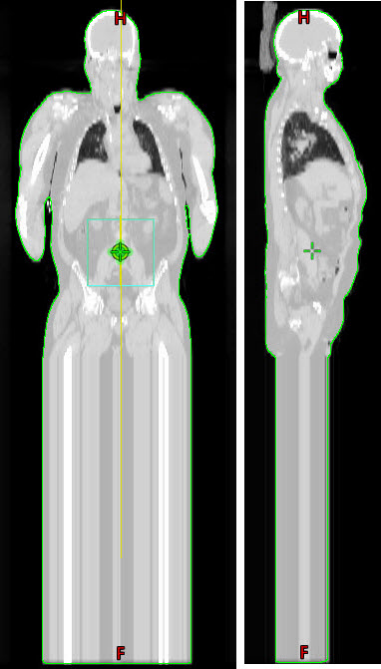
Example of a patient CT scan with the mid‐thigh position extended to the height of the patient.

A treatment planning CT scan was acquired for each patient. Patients are setup supine with supports under the knees and feet and with hands resting on thighs. Measurements of the patient length, knee separation, ankle separation and arm strap length were recorded. A CT scan of 135 cm in length was taken (0.5 cm thick slices); for adult patients this encompasses the top of the head to approximately middle thigh. Patients are setup prone to confirm that the position will accurately represent a flipped version of the supine setup. This is done by confirming that the patient's back is horizontal and that all the measurements taken during the supine setup match. Using the Eclipse^®^ TPS (Varian Medical Systems), an extended CT scan, equal to the patient's height, was created by replicating a slice from midthigh to the correct length (Fig. 1). In addition to the Body contour, additional structures were created: Body retracted by 5 mm from skin (*Body‐5 mm)*, a Flash structure consisting of a 3 cm rind around the Body (*Flash)*, both lungs in a single contour (*Lungs*), and a calculation volume including all of the structures (*Calc Volume*).

#### 2.B.2 Treatment planning

##### Standard plans

**Figure 2:**
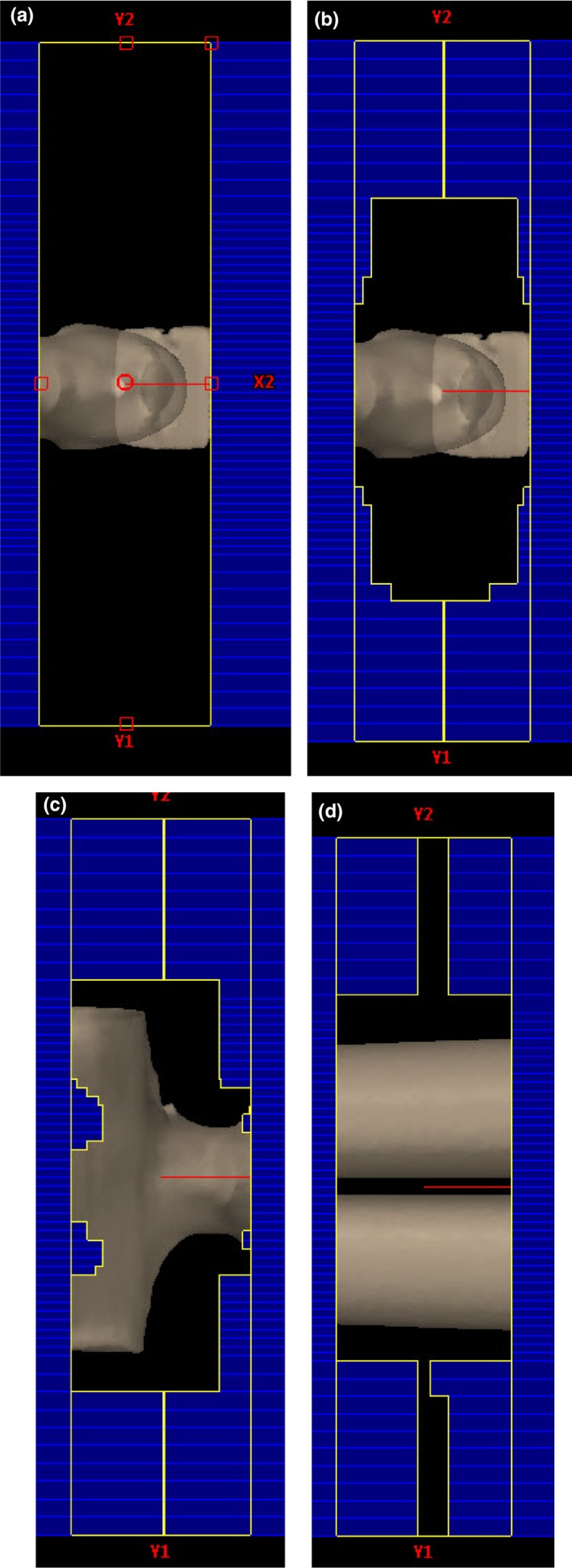
Beams eye view (a) From an open field standard plan at the patient's head, before optimization; (b) from an optimized plan at the patient head; (c) from an optimized plan at the patient chest; (d) from an optimized plan at the patient legs.

Custom VMAT arcs, ranging from 310° to 60° were created with a static 10 × 40 cm^2^ opening (Fig. 2a). Plan meterset weights were altered using custom Python code to deliver more MUs at the periphery of the arc to account for the ISL effects.^20^ Standard meterset weights were determined based on Jahnke et al.'s work, and adjusted after testing dose homogeneity using dose calculated on patient CT scans (Table 1). Calculated meterset weights were spread over 91 control points. The treatment plan dose was calculated with the couch set to 90° using 7–8 arcs for the prone and supine orientations separately. Monitor units were adjusted to ensure full D98 coverage of the *Body‐5 mm*. For a 2 Gy/fx plan, this resulted in ~700 MU per arc, a midplane instantaneous dose rate of ~1.0–1.3 Gy/min, and an average dose rate of each arc at midplane of ~0.10–0.12 Gy/min.

**Table 1:** Meterset weights and resulting gantry speeds based on 700 MU

Angle (°)	Meterset weight	Gantry speed (°/sec)
310–320	2.250	1.071
320–330	1.750	1.377
330–340	1.229	1.961
340–350	1.074	2.245
350–10	1.000	2.411
10–20	1.074	2.245
20–30	1.100	2.191
30–40	1.277	1.880
40–50	2.000	1.205
50–55	2.667	0.904
55–60	3.780	0.638

##### Optimized plan

Multi‐leaf collimator modulation was used to reduce the dose to the patient's periphery and lungs. MLC positions were obtained using the VMAT optimizer provided by the Eclipse^®^ TPS (version 11.0.31). The patient supine plan with dose calculated from the standard plan arcs was opened in the optimizer. Optimization was limited to the last step of the VMAT Progressive Multi‐resolution Optimizer (PRO step 4/4). Through the use of only the last PRO step only minor changes to the standard plan are made, limited to the MUs and MLC position (Fig. 2b–d). Optimization parameters are chosen to maintain coverage to the Body‐5 mm contour and Flash, while limiting dose to the lungs to the prescription dose. The entire prescription dose was optimized by including the prone plan as a base dose plan. The optimizer takes approximately 5–10 minutes to converge on a solution, and the process was then repeated for the prone plan using the supine plan as a base dose plan. Once optimization was completed the MUs were adjusted by the spoiler correction factor (SCF) (3% higher). Treatment planning time requires 90–120 minutes.

Patient‐specific QA is performed for one supine and one prone arc from all patient plans. This is done through delivery of an EPID on the treatment unit used for treatment. Plans are analyzed for 95% pass rates at 3%/3 mm.

#### 2.B.3 Treatment delivery

**Figure 3:**
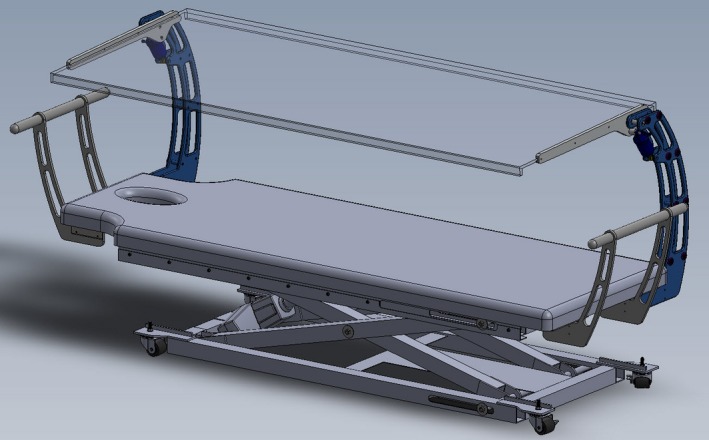
CAD drawing of the converted massage table with beam spoiler used for treatment.

Treatment was delivered with the patient setup on a modified massage table capable of lowering the patient to a position of 175 cm SSD. The bed is setup perpendicular to the conventional couch and modified to have a 1 cm thick acrylic spoiler that traverses the entire length of the patient (Fig. 3). Both the spoiler and the bed can be raised and lowered for ease of patient transfer.

Typical treatment workflow is as follows:

1. The bed is setup at 90° and then centered and aligned with the in‐room lasers. (Fig. 4)

**Figure 4:**
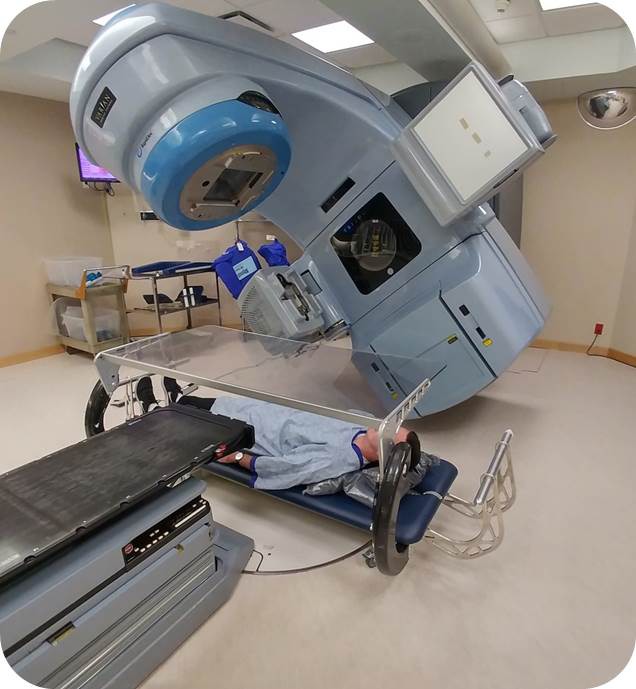
Photo of the treatment bed, in position for treatment with patient laying supine. Treatment beam is directed toward patient head.
2. The patient lies on the bed. With the gantry at 0°, the crosshair is aligned to the umbilicus, the patient is raised/lowered to the correct SSD, and the spoiler is lowered.
3. The treatment plan is *Moded‐Up* and the patient position is confirmed using the MLC shaped light field at numerous gantry angles.
4. In vivo verification diodes and OSLDs are placed at four sites (head [temple], umbilicus, lumbar back behind umbilicus and the top of the right foot).
5. The plan is delivered and diode readings are confirmed to match the dose predicted from the TPS for each arc.
6. The patient is then setup prone and the procedure is repeated.

The entire process takes approximately 45–60 minutes for the first fraction and less for subsequent fractions.

### 2.C Shift analysis

To assess the plan robustness to setup uncertainties, the dosimetric effect of setup uncertainties was tested by systematically shifting the CT scan in crainial‐caudal (CC), lateral (Lat) and anterior‐posterior (AP) directions and recalculating the dose. The shifts were applied to both the prone and supine plans in the same direction to simulate the maximum error. Simulated shifts were +1 and +2 cm in the Lat and CC directions and ±1 and 2 cm in the AP direction (simulating SSD mismatch). Simulated setup uncertainties were chosen to be multiple times larger than uncertainties generally seen in patients that are setup with minimal immobilization.^17^ Plans were compared to unshifted plans for dosimetric consequences. This was tested for 15 patients and the results of D98% coverage and D2% hot spots to the Body‐5 mm was reported, as well as the change to MLD.

### 2.D Treatment beam verification measurements

A series of measurements at extended SSD were performed to ensure that the TPS correctly models the machine output and beam profiles at these distances. These measurements are not typically performed at commissioning time and are required for extended SSD treatments.

The first measurement was made to verify the ability of the TPS to model the absolute output (cGy) as a function of depth at extended SSD for a fixed field. A 10 × 10 cm^2^ field size was used to isolate the output measurement from field size effects. A C11 Ion Chamber (Capintec, Florham Park, NJ, USA) was placed in a 30 × 30 × 30 cm^3^ solid water phantom at an SSD of 175 cm. Depth dose measurements were made at depths of 5, 10, 15, and 20 cm for a delivery of 500 MU at each depth. A predicted dose from the TPS was modeled by creating a digital water phantom of the same size. Percentage differences for each point are reported.

Inline profile measures were made at an SSD of 175 cm at depths of 1.5 (~*d*max), 5 and 10 cm. Measurements were made using an IC Profiler^®^ (Sun Nuclear Corporation, Melbourne, FL, USA) at three different in‐line positions and the profiles were stitched together. A field size of 10 × 40 cm was used to correspond to the field size used clinically. Measurements were repeated with a 1 cm thick acrylic spoiler to determine the effect of the spoiler because it is not modeled. Both sets of measurements were normalized to the central detector and compared to predicted dose profiles from the TPS. Maximum and mean dose differences within the central 80% of the fields are reported.

A set of measurements was performed to validate the ability of the TPS to model the delivery of dynamic fields at extended SSD (175 cm). Fields obtained from optimized patient plans were used to ensure the amount of modulation being tested was accurate; all fields were 10 × 40 cm^2^ and delivered from 310 to 60 degrees. Absolute point dose measurements were made with an ion chamber at 10 cm depth in a 30 × 30 × 30 cm^3^ solid water phantom at three separate longitudinal locations. The SCF was determined by remeasuring these fields at the three separate points with the 1 cm acrylic spoiler in place. The average ratio of these measurements was taken to determine the percentage increase in MUs that must be used to obtain the same midplane dose with the spoiler present.

A representative AP field from a patient plan was chosen on which to perform multiple 2D film measurements. 2D field profile measurements were obtained using a piece of Gafchromic^®^ film (Ashland^™^, Bridgewater, NJ, USA) at a depth of 10 cm in a 30 × 30 × 30 cm^3^ phantom at four separate longitudinal locations. Locations were chosen to model the head, lungs (film only), umbilicus, and feet. Ion chamber point dose measurements were made for fields from five separate patients; Gafchromic^®^ film measurements were made for a field from one patient. Measurements were compared to the dose predicted from the TPS, by recalculating the patient treatment plan on a virtual water phantom. The film was calibrated using a wedge calibration factor, and they were compared to dose planes exported from the TPS. Gamma analysis was performed and pass rates are reported for 5%/1 mm.

A final set of measurements were made to determine the surface dose delivered with the spoiler in place and the SCF applied. This is required as the spoiler is not modeled in the TPS. A treatment plan consisting of both supine and prone arcs was developed to deliver 200 cGy in one fraction to an Alderson phantom (Radiology Support Devices, Long Beach, CA, USA). Strips of Gafchromic^®^ film (20 cm × 6.25 cm) were taped to the Alderson phantom's surface on both the front and back over multiple locations. The films were prescanned with an Epson Expression 10000XL scanner in order to correct for non‐uniformities. Absolute dose was determined using a wedge calibration matched to a premeasured dose profile using DoseLab Pro^®^ (Mobius Medical Systems, Houston, TX, USA). The measured dose was compared to the expected prescription dose. The full plan was delivered as per the protocol outline in the treatment delivery section.

## 3 RESULTS

### 3.A Patient dosimetric results

**Figure 5:**
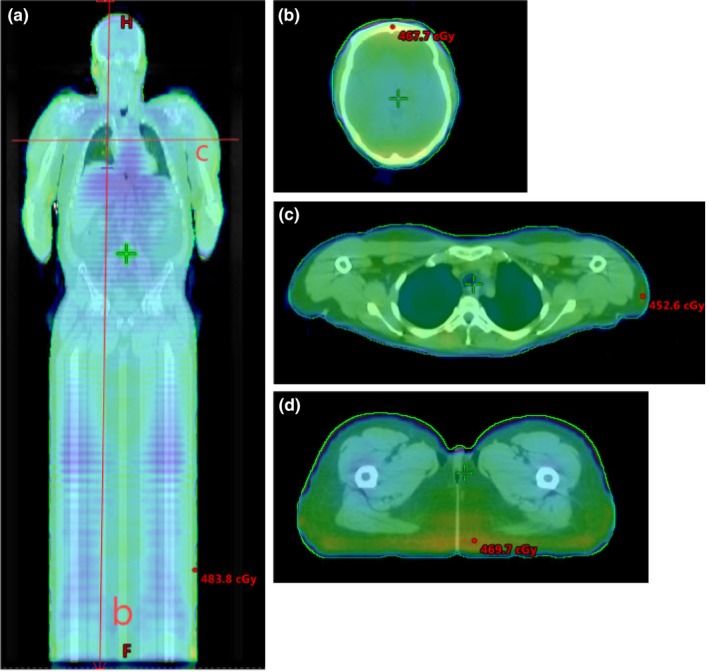
(a) Coronal dose colorwash image from an optimized patient plan. The dose profiles indicated are shown in Fig. 6; (b) axial color wash dose image from the head; (c) axial color wash dosed image from the chest; (d) axial color wash dose image from the legs.

**Figure 6:**
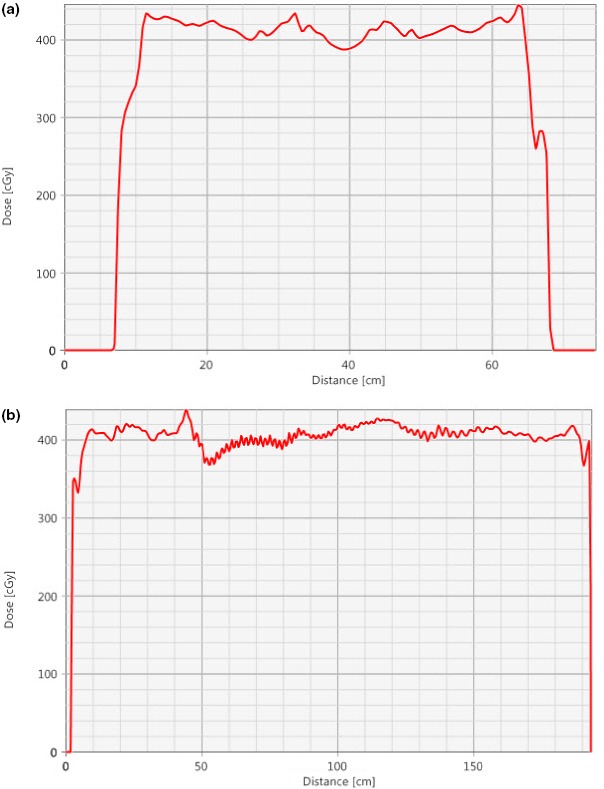
(a) Dose profile across the chest as indicated in Fig. 5a; (b) dose profile from head to legs as indicated in Fig. 5(a). Both dose profiles exhibit the homogenous dose to the patient at a prescription of 400 cGy.

**Figure 7:**
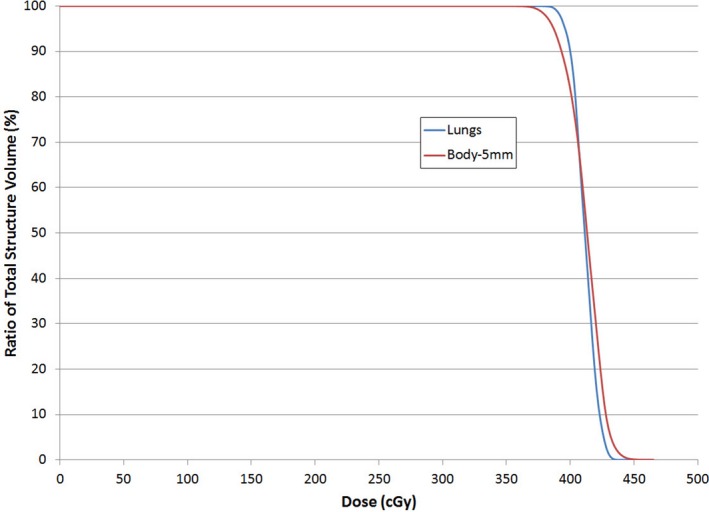
Dose volume histogram for a representative patient. Both the target (Body‐5 mm) and the Lungs are shown, and obtain the prescription dose (400 cGy) with a sharp falloff.

**Figure 8:**
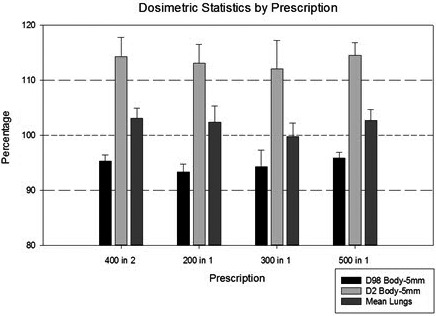
Chart of the three dosimetric parameters used to assess our treatment plans, presented as a function prescription dose. Error bars are two standard deviations.

Multiple slices from a patient CT scan with dose in color wash are shown in Fig. 5. Two dose profiles are shown, one down the midline and one across the lungs (Fig. 6). These profiles illustrate a homogenous dose within ±10% of the prescription coverage of the Body‐5 mm structure, and the MLD was recorded from the plan sum of the supine and prone plans for all 109 patients. A representative dose volume histogram including the Body‐5 mm contour and the Lungs contour is shown in Fig. 7. Results are presented as a percentage of the prescription dose in Fig. 8. The largest percentage of patients receives 400 cGy in two fractions (86/109), and their coverage was consistently over 95%. MLD was limited to approximately 100% of the prescription dose. The D2% hot spot was greater than our target of 110%, however the difference between the D98% and the D2% was consistently <20%.

**Figure 9:**
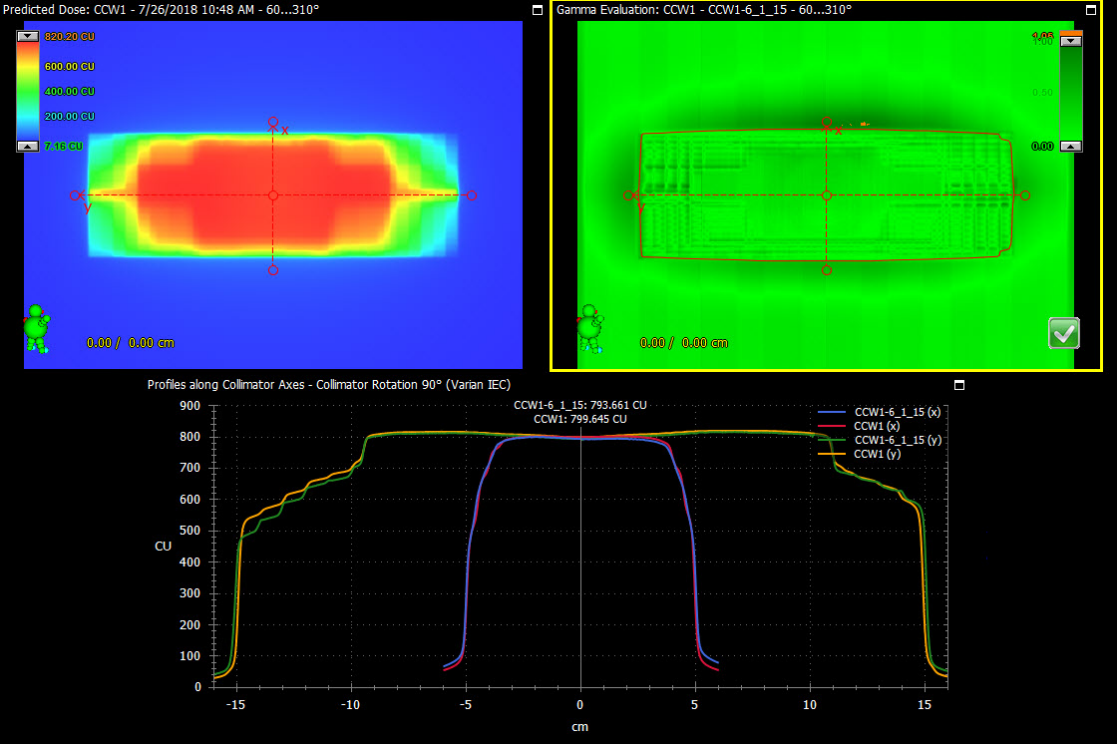
Image of a delivered EPID treatment verification plan (top left), the gamma map at 3%/3 mm between the delivered and planned images (top right), and profiles comparing the two images (lower).

Results of OSLD measurements from five patients for the standard eight OSLD sites (head [temple], belly button, back, foot and four leg positions) are shown in Table 2 with a comparison to a single fraction dose of 200 cGy. Our tolerance for these measurements is ±10% for the morning fractions. If this is exceeded, we would consider a change for the afternoon. To date, this has not occurred. An example of the patient specific QA is shown in Fig. 9. The pass rate at 3%/3 mm is 99%, which is typical for these plans. Of note is how flat the dose profile is, due to the small amount of modulation present.

**Table 2:** Results of OSLD In Vivo Measurements

Location	Value (cGy)	Range (cGy)	% Difference from 200 cGy
Belly button	209.6	202.9–218.4	4.8
Head (Temple)	215.1	201.3–231.8	7.6
Back	209.4	200.1–216.7	4.7
Foot	199.6	192.3–209.9	−0.2
Anterior thigh	188.5	182.0–197.1	−5.8
Posterior thigh	194.3	185.0–202.7	−2.8
Anterior calf	198.4	183.8–212.0	−0.8
Posterior calf	199.4	189.3–215.2	−0.3

### 3.B Shift analysis

Shifts in planned setup were simulated in the TPS and compared to the planned dose. Both Lateral and Superior–Inferior shifts showed robustness to change with values <1% for a 1 cm shift and only the hot spot (D2) showing a value larger than 1.1% for a 2 cm shift (3.8% for the Lateral shifts). The Anterior and Posterior shifts were made to simulate an SSD mismatched. These values were generally larger than the other shifts, however an SSD change of 2 cm showed a maximum of 4% change in the parameters; this occurred in the hot spot. A summary of all the shift differences performed are shown in Table 3.

**Table 3:** Summary of DVH Parameters after Shift Tests

Direction	Parameter	No shift (Gy)	Shift 1 cm		Shift 2 cm	
			(Gy)	(% Change)	(Gy)	(% Change)
Lateral	D98	3.83	3.83	−0.1	3.81	−0.6
	D2	5.00	5.04	0.8	5.18	3.8
	MLD	4.15	4.15	0.1	4.17	0.5
Superior‐inferior	D98	3.83	3.83	0.0	3.82	−0.2
	D2	5.00	5.00	0.0	5.00	0.1
	MLD	4.15	4.17	0.5	4.19	1.1
Anterior	D98	3.83	3.77	−1.5	3.71	−3.2
	D2	5.00	4.93	−1.4	4.84	−3.2
	MLD	4.15	4.09	−1.3	4.03	−2.8
Posterior	D98	3.83	3.90	1.7	3.96	3.5
	D2	5.00	5.09	1.9	5.19	4.0
	MLD	4.15	4.21	1.6	4.28	3.3

### 3.C Treatment beam verification measurements

**Figure 10:**
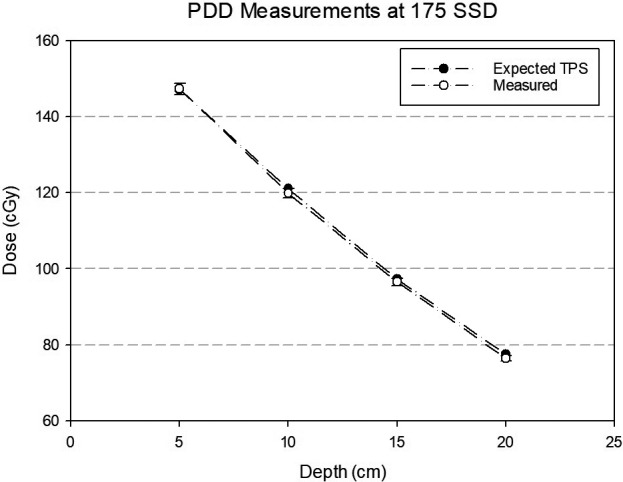
Absolute depth dose curve at extended SSD comparing measurements (CC13 chamber) to the same geometry in the treatment planning system. Error bars are 1%. Simulated and actual measurements were in solid water.

A set of 4 measurements to verify the percentage depth dose in a solid water phantom at an extended SSD of 175 cm are shown in Fig. 10. The expected dose from the TPS is plotted alongside the measurements. All values showed excellent correspondence, with the worst agreement occurring at a depth of 20 cm (−1.4%).

**Figure 11:**
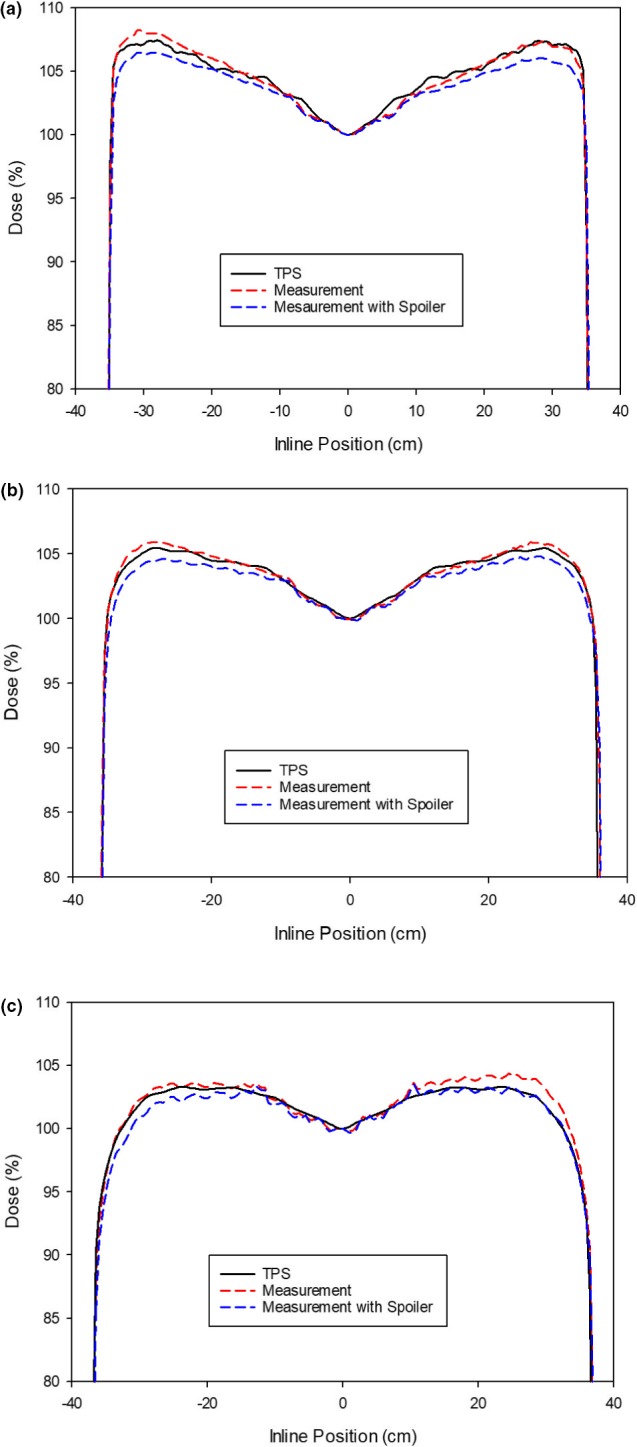
Inline profiles obtained using the ICProfiler comparing predicted and measured field at three separate depths: (a) 1.5 cm; (b) 5 cm; (c) 10 cm. Profiles show good correspondence with and without the beam spoiler.

Inline profiles were measured for a static 10 × 40 cm^2^ field at 175 cm SSD at three separate depths. Profiles were measured both with and without the spoiler present. The normalized data are shown in Fig. 11(a–c) for each of the three depths along with the data from the TPS. The maximum and mean percentage differences were measured within the central 80% of the beam for all profiles versus the TPS's profiles. All mean percentage difference values were <1%, with the maximum deviations <1.3%. Summary values are shown in Table 4.

**Table 4:** Summary of Profile Comparisons

Profile depth	Spoiler	Absolute maximum difference (%)	Average difference (%)	Standard deviation (%)
dMax (1.5 cm)	No	1.1	0.1	0.4
5 cm	No	0.4	0.0	0.2
10 cm	No	1.2	0.3	0.4
dMax (1.5 cm)	Yes	1.3	0.6	0.3
5 cm	Yes	1.0	0.5	0.2
10 cm	Yes	1.1	0.3	0.3

Point dose measurements at three separate points along the crossline axis were made in solid water at a depth of 10 cm with a Capintec C11 ion chamber. The difference in point dose values from the TPS predicted values (average, [range]) at the locations to simulate the head (70 cm superior), umbilicus (0 cm), and feet (90 cm inferior) were 2.9% (−5.2%, 0.4%), 0.0 (−0.4%, 0.3%), and −0.3% (1.8%,3.5%). The measured values show excellent correspondence to the predicted values.

The same set of point dose measurements were re‐measured with the 1 cm Lucite spoiler in place. The percent difference between the no‐spoiler and spoiler plans were averaged to give a value of 4.0% (3.0%, 5.1%). This value is applied to all plans to account for the spoiler attenuation of the beam.

**Figure 12:**
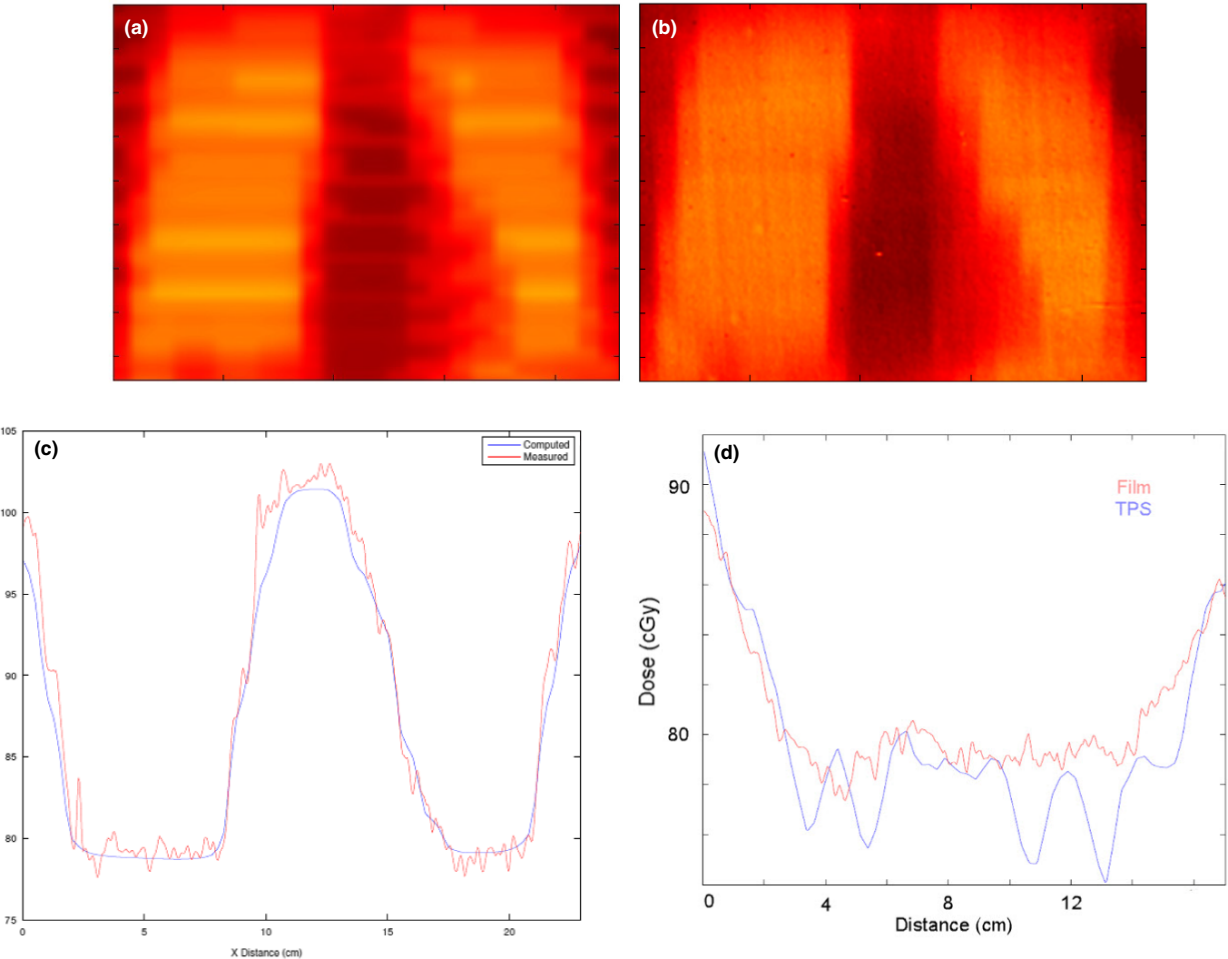
Film analysis example shows the predicted distribution (a) and the measured distribution (b) at the location of the lungs. Lateral (c) and superior‐inferior (d) dose profiles show excellent correspondence between the measured and predicted values.

2D film analysis of the representative AP patient arc results in gamma pass rates, at 5%/1 mm, with respect to corresponding superior‐inferior locations, of 95.3% (+55 cm; head), 97.6% (+35 cm; lungs), 99.5% (0 cm; abdomen), and 91.5% (−55 cm; legs). A more in‐depth examination of the TPS predicted dose and film dose from the section occurring in the phantom's “lungs” is shown in Fig. 12. Presented with the planar images of the predicted dose and measured film are profiles taken in the two directions of the film. The profile through the lateral direction (Fig. 12 c) shows excellent correspondence between the two scans. The crainial‐Caudal profile (Fig. 12 d) displays the limitation of the TPS to calculate VMAT arcs at extended SSD—specifically the sinuous appearance created by calculating the dose only at controls points, not between control points. This pattern did not appear to greatly influence the pass rate of the film; however, it is likely contributing to the smaller pass rate seen in the leg film.

Results of the eight films placed on the surface of the Alderson phantom are shown in Table 5. Multiple ROI were taken to assess different regions. All doses were above 175 cGy (87.5% of prescription dose), confirming the SCF and the use of the spoiler to promote skin dose.

**Table 5:** Summary of Surface Dose Comparisons

Location	Average dose in ROI (cGy)	SD (cGy)
*Supine*		
Front of head left	181	2
Front of head right	182	3
Chest	208	6
Belly	191	2
Left thigh	178	3
Side of left thigh	217	4
*Prone*		
Back of head	176	11
Back shoulders	203	7
Mid back	195	3
Right buttocks	189	11

## 4 DISCUSSION

In this work, we present a new TBI delivery technique that uses MLC shaping to accomplish dose homogeneity. The use of a modulated gantry speed addresses ISL changes for arc delivery, similar to the technique presented by Jahnke et al.^20^ The optimizer from Eclipse^®^ is used to shape the MLC and adjust the MUs of each arc. Using only the final step of the PRO we maintain the set meterset weights for each control point while altering the total MU per arc and the MLC positions. The standard beam model using the AAA 11.0.31 calculation algorithm is used to calculate the dose. Employing a commercial beam optimizer ensures a plan's deliverability and permits the use of the record and verify system, which is an integral part of radiation therapy treatment delivery and not always available for TBI treatments.

For the dose calculation, a flipped supine scan is used as the prone scan to calculate the dose. This was done for practical considerations, such as the ability to dose sum without using nonlinear registration. Patients are setup both prone and supine during CT scanning to ensure that the positions are reproducible. To confirm that this assumption provides a minimum amount of setup uncertainty, prone CT scans were taken for the first year this technique was in use (approx. 100 patients). For each patient, the dose was calculated on both the flipped supine and prone scan and compared. Minimal differences were found between the two scans. Additionally, for a subset of patients deformable image registration was performed in order to produce a plan sum between the prone and supine scan. Again, minimal differences were found giving confidence that the flipped supine scan can be used to represent the prone scan. Further support for this assumption is based on the emphasis on the use of low modulation for these plans to ensure setup uncertainties had small effects on the overall plan coverage. As the results of the shift analysis have shown, lateral and superior–inferior shifts up to 2 cm have minimal effect on the overall dose distribution.

Patient setup is done without the use of IGRT technology, which is currently the standard of practice for inverse optimized treatment plans. It was speculated that the modulated MLC and gantry speed motion would be potential sources of error if the patient setup was different during treatment in comparison to the CT simulator. Using cross hairs, a custom ruler, lasers, and the treatment field light we are confident that our patients are setup within 1 cm of the scanned position based on published values of setup uncertainties with minimal immoblization.^23^ To ensure that potential motion is taken into account, a 3 cm uniform flash structure is added for planning. This technique combined with minimal MLC motion resulted in the treatment plan's insensitivity to motion, which was tested and verified through a shift invariance study.

The instantaneous dose rate of this technique is significantly higher than is conventionally used for TBI, while the average dose rate is within range of what has been used by other studies. Dose rate concerns are primarily due to increased risk of radiation induced pneumonitis. This risk is reported at higher TBI doses than we use in this study.^3^, ^4^ At low doses there have been limited reports of lung injury in patients without comorbidities. It is our aim to further investigate dose rate concerns as the use of FFF beams would significantly reduce treatment times and promote patient comfort.

Our previously used technique (lateral POP beam under full bolus) required the use of anesthetic for select patients with claustrophobic concerns and for pediatric patients. Patients requiring anesthetic are not treated with the technique presented. However, a large number of patients that would usually require anesthetic have been treated without anesthetic using our new technique. Consequently, only infants are now anesthetized. In addition, our new setup has allowed for pediatric patients to be treated while viewing an iPad^®^ (Apple, Cupertine, CA), which is placed out of the beam resting on the spoiler for the supine orientation and below the bed for the prone orientation. The iPad serves as an immobilization device, and has allowed us to treat a patient as young as 5 years old.

The low dose TBI regimen used at our center does not necessitate significant shielding to organs at risk, like the lung or kidneys. However, the use of higher doses often seen in TBI (eg 12 Gy in 6 fractions) would require significant shielding. We have tested our technique for a common TBI prescription of 12 Gy in six fractions, and have been able to achieve a lung dose as low as 6 Gy. The modified technique relied on manual manipulation of the MLCs and a slightly smaller field size (10 × 40 cm^2^), but the results were promising.

## 5 CONCLUSIONS

In this work, we presented a TBI technique that delivers at extended SSD and provides organ at risk shielding with minimal MLC modulation. The workflow is completed entirely using commercial treatment planning software, ensuring deliverability and consistency. The method has been implemented for over 100 patients at our center. Dosimetric verification measurements were performed prior to technique implementation and showed that separate beam model data was not required. Data measured at extended SSD match predicted data from the TPS. This technique has been shown to be robust and patient sensitive, while provided a safe treatment that utilizes both the record and verify system and the commercial TPS.

## CONFLICTS OF INTEREST

The authors have no relevant conflicts of interest to disclose.
